# Prefrontal and parieto-occipital neural signatures of evidence accumulation and response to computerised Cognitive Behavioural Therapy in depression

**DOI:** 10.1038/s44184-025-00165-3

**Published:** 2025-10-14

**Authors:** Filippo Queirazza, Marios G. Philiastides

**Affiliations:** 1 School of Health and Wellbeing, University of Glasgow, Glasgow, UK; 2 School of Psychology and Neuroscience, University of Glasgow, Glasgow, UK

**Keywords:** Decision, Learning algorithms, Prognostic markers, Depression

## Abstract

Computerised Cognitive Behavioural therapy (CBT) is an effective psychological intervention for mild to moderate depression. While CBT aims to correct maladaptive cognitive biases and ensuing disadvantageous decision-making, our current understanding of decision-making signatures linked to CBT response remains limited. Preliminary behavioural evidence has shown that the process of evidence accumulation (EA), indexing the efficiency of decision dynamics, is impaired in depression. However, little is known about the role of EA in the context of CBT for depression. In this study we recruited 37 (18 females) unmedicated depressed subjects. Participants attended two task-based functional resonance imaging sessions before and two months after completing an online self-help CBT-based intervention. We fitted a hybrid reinforcement learning drift diffusion model to the probabilistic reversal learning task data and investigated accumulator-like brain activity as a function of response to computerised CBT. We found that at baseline, compared to nonresponders, responders exhibited weaker left prefrontal and parieto-occipital EA neural signatures, which subsequently increased in proportion to the sustained symptomatic improvement observed following computerised CBT. We thus provide preliminary evidence that attenuated EA neural signatures in the left prefrontal and parieto-occipital cortical areas are associated with response to computerised CBT in depression. Crucially, the observed increase of accumulator-like brain activity following computerised CBT warrants further replication in future experimental work probing neurocomputational mechanisms of change in CBT.

## Introduction

Cognitive behavioural therapy (CBT) is an evidence-based treatment for depression^1^, with well-established effectiveness^2,3^. Computerised CBT (cCBT), which is Internet-delivered self-help CBT, is recommended as first-line treatment option for mild to moderate depression^1^. Notably, cCBT is cost-effective, widely accessible, and acceptable to patients^4,5^.

CBT aims to correct the negatively biased acquisition and processing of information, which are the core cognitive dysfunctions characterising depressive disorder^6^. While distorted inference and subsequent maladaptive decision-making are the critical targets of cognitive restructuring, one of the core therapeutic components of any CBT-based intervention^6^, a detailed understanding of the decision-making signatures associated with CBT response is still lacking.

Previous fMRI work has linked pre-treatment activity in the anterior cingulate cortex^7–11^, amygdala^8,12^, prefrontal cortex^13^ and striatum^12^ to CBT response in depression. Neuroimaging predictors of cCBT response would have significant implications for enabling effective personalised treatment plans, optimising resource allocation, and addressing treatment inequalities in healthcare.

In recent years the application of computational approaches to psychotherapy research has furnished a new theoretical framework whereby latent neurocognitive decision processes can be explicitly formalised in mathematical terms and meaningfully linked to psychotherapy^14,15^. Importantly, computational methods can also help elucidate how distinct decision processes are realised at the neural level. Accordingly, computational models can inform the analysis of neuroimaging data and enable identification of theoretically grounded and interpretable neural signatures of psychotherapy response^12^.

Within the computational framework of stochastic diffusion models, bounded evidence accumulation (EA) is theorised to represent a fundamental cognitive process underpinning decision-making under time pressure and refers to the gradual accumulation of noisy information until a threshold of evidence is reached and a decision is made^16,17^. Diffusion models have been successfully employed to account for the observed speed and accuracy of sensorimotor decisions^16,17^. These models assume that decision evidence is integrated over time up to a decision threshold and this process is subject to random noise^18^.

The seminal findings that the firing rate pattern of neurons in the frontal eye fields^19^, lateral intraparietal cortex^20–23^ and dorsolateral prefrontal cortex^24^ of non-human primates closely mirrored the gradual and noisy accumulation of (perceptual) evidence to a fixed (decision) threshold, provided impetus for the widespread application of diffusion models to unlocking the neural basis of perceptual decision-making^25^.

Crucially, while EA neural signatures were initially uncovered in the context of non-human^19–24^ and human^26–38^ perceptual decisions, they have now been linked to value-based choice behaviour and associative learning^39–42^, thus emerging as a domain-general decision-making process^41,43,44^. The rate of EA (also known as drift rate) reflects the discriminability (that is, signal to noise ratio or SNR) of decision evidence, which is a function of decision difficulty and individual ability to discern decision-relevant information^17^. As such it is often used as an index of the quality of the evidence entering the decision process and by extension, the overall efficiency of decision dynamics; greater values of the drift rate indicate more efficient information processing^17^.

Relative to control conditions, smaller estimates of the drift rate denoting sluggish EA have been linked to depression^45–48^, schizophrenia^49^, ADHD^50–52^ and the global severity of psychopathology across different mental disorders^53^, underscoring its potential utility as a task-general, transdiagnostic vulnerability factor^43^. Recently we showed that in healthy subjects, experimentally induced mild inflammation, of magnitude similar to that observed in depression^54,55^, diminishes EA signatures recorded using functional resonance imaging (fMRI)^42^. To the best of our knowledge, no prior study has probed the neural efficiency of EA processes in the context of psychotherapy for depression.

To bridge this gap, we examined behavioural, and fMRI data collected from a sample of unmedicated depressed subjects before and two months after completing cCBT^12^. Using a hybrid reinforcement learning drift diffusion model (RLDDM) to describe choice selection and latencies elicited during a probabilistic reversal learning (PRL) task, we investigated fMRI activity parametrically scaling with EA (more precisely, the trial-wise amount of integrated decision evidence) at the time of choice. We reasoned that, since CBT works by targeting negatively biased cognitive appraisal and by extension, maladaptive decision-making, a key decision-making signature like the efficiency of evidence accumulation (and its neural underpinnings) would play a central role in enabling CBT response and be ameliorated by successful CBT treatment. We capitalised on the haemodynamic responses denoting gathering of decision evidence in prefrontal and parieto-occipital accumulator regions of the brain to differentiate between cCBT response groups and classify individual cCBT response. Moreover, we explored whether post-treatment change of the identified accumulator-like pattern of blood-oxygen level-dependent (BOLD) activity was significantly correlated with symptomatic improvement.

In conclusion, our work provides preliminary evidence that attenuated prefrontal and parieto-occipital EA fMRI signatures indexing diminished neural efficiency of evidence integration are associated with cCBT response. These findings add to previous research on the candidate neurocomputational assays of response to psychotherapy and their validity and clinical utility as predictive biomarkers could be further tested in future experimental work.

## Methods

### Study design and intervention

Participants were recruited as part of the Glasgow CBT study (see Supplemental Information for more details on this). Participants attended a baseline appointment before cCBT and a follow-up review two months after completion of cCBT to evaluate sustained response to cCBT. A clinical diagnosis of depression was confirmed using the Clinical Interview Schedule-Revised (CIS-R)^56^ and depression severity was measured using the Beck’s Depression Inventory-II (BDI-II)^57^. Response to cCBT was defined as a ≥50% reduction in the pre-treatment BDI-II score and noncompletion of cCBT was regarded as an index of treatment failure. Based on an effect size (i.e., d = 0.42) determined by performing an ROI analysis^58^ on a similar task-based fMRI dataset^42^, we estimated that a sample size of 37 subjects would be powered enough (i.e., 80% at alpha = 0.5) to retrieve reliable accumulator-like activity in the brain. All participants provided written informed consent, and the study protocol was approved by the West of Scotland Ethics Committee (10/S0703/71).

### Behavioural task and analysis

**Fig. 1:**
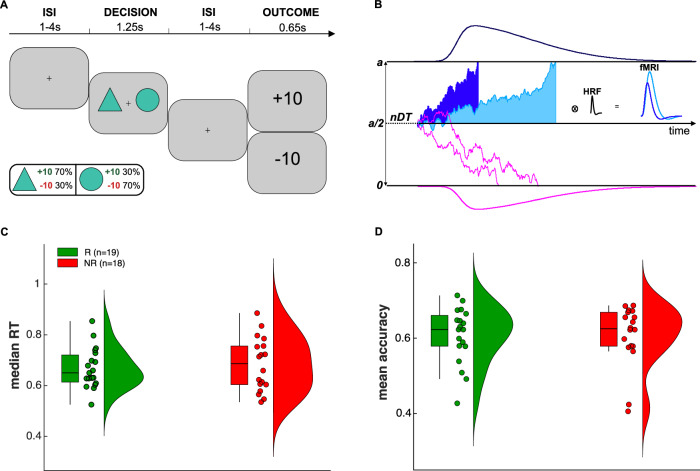
Task, computational model and behavioural results. **A** Probabilistic reversal learning (PRL) task. At the beginning of each trial participants were shown a fixation cross of jittered duration (between 1 and 4 s) followed by two geometrical shapes, which were sampled from a pool of 18 different geometrical shapes and appeared randomly on either side of the screen for 1.25 s. Participants had 1 s to make a choice via a button press. In case of late responses, they were prompted to respond faster. The outcome of their decision was displayed for 0.65 s after a second jittered interstimulus interval and was either positive (+10) or negative (−10). As shown in the box, the reinforcement schedule was probabilistic and asymmetrically skewed so that one stimulus (i.e. the high probability stimulus) was more likely to be associated with a positive outcome than the other stimulus (i.e. the low probability stimulus). Crucially, reinforcement contingencies were reversed based on a fixed learning criterion. We also added a randomly generated number of buffer trials before reversals to prevent participants from easily figuring out the underlying learning criterion. Prior to the experiment, participants were advised of the probabilistic nature of the task and that reinforcement contingencies might reverse based on their performance. They were allowed to practice the task for 5 min to become familiar with the speed requirements of the task prior to fMRI scanning. Moreover, they were advised they would be remunerated based on their task performance. ISI stands for interstimulus interval. **B** The choice rule of the computational model was a pure drift-diffusion process. Solid EA traces represent moment-by-moment ramping up of decision evidence at fast (blue) and slow (light blue) accumulation rates. The shaded areas under the EA ramps denote the amount of integrated decision evidence. Convolving a greater amount of integrated evidence with the haemodynamic response function (HRF) yields increasingly higher predicted peak BOLD responses. nDT stands for nondecision time. Raincloud plots showing pre-cCBT between-group comparisons of median RTs (**C**) and mean choice accuracies (**D**) recorded during the PRL task. Boxplots display median and interquartile range. Colour-coded dots represent responders (R, green) and nonresponders (NR, red).

We used the PRL task (*n* = 180), which was previously described in refs. ^12,59,60^ and is shown in Fig. 1A. To investigate differential effects of cCBT response on choice accuracy and latencies during the behavioural task, we conducted maximal by-subject generalised and loglinear mixed-effects models using the *lme4* package in R (http://www.r-project.org) and allowing for random correlations between the independent variables^61^. We tested the statistical significance of the fixed effects using the likelihood ratio test^61^.

To test for between-group (that is, responders versus nonresponders) differences in response times (RT) pre- (Eq. (1)) and post-cCBT (Eq. (2)), we ran the following mixed-effects loglinear regression models:

1 $$\log \left(R{T}_{{bsln}}\right)=1+{response}+{preBDI}+(1{|subject})$$

2 $$\log \left(R{T}_{{all}}\right)=1+{response}* {session}+{preBDI}+(1+{session|subject})$$

where *response* and *session* are categorical variables indexing cCBT response (i.e., responders versus nonresponders) and study’s visit (i.e., pre- versus post-cCBT). The interaction term *response:session* captures any significant between-session RT changes as a function of cCBT response. The *preBDI* variable is a nuisance covariate accounting for behavioural effects primarily associated with pre-treatment severity of depressive symptoms as measured by BDI-II z-scores.

Moreover, to examine any differential effect of feedback valence (*fbk*_*val*_) and choice accuracy (*acc*) on RTs as a function of cCBT response, we performed the following regression analyses:

3 $${\text{log}}\left({RT}\right)=1+{fb}{k}_{{val}}* {response}+{preBDI}+(1+{fb}{k}_{{val}}{|subject})$$

4 $$\log \left({RT}\right)=1+{acc}* {response}+{preBDI}+(1+{acc}|{subject})$$

We also assessed for between-group differences in choice accuracy pre- and post-cCBT.

### The hybrid RLDDM model

To dissect the neurocomputational underpinnings of decision dynamics during the PRL task we devised a hybrid RLDDM model^62,63^ that combines cognitive processes underlying feedback-driven learning and value-based decision making. The choice rule of the RLDDM model is a drift-diffusion process, which accounts for both choice accuracy and the speed-accuracy trade-off (see ref. ^64^ for a primer on the drift-diffusion model). According to the DDM framework, decision evidence is continuously and stochastically integrated until one of two termination boundaries is reached, leading to a response. Boundary separation is modelled with a parameter *a*, which implements the speed-accuracy trade-off and is thus an index of response caution. Wider decision bounds are typically associated with slower decision times and more accurate decisions (as there is more time to average out random noise). Conversely, narrower decision bounds lead to faster decision times but less accurate decisions (as there is less time to average out random noise)^18^. Non-decision time processes including stimulus encoding and motor preparation/execution are captured by an additional parameter *nDT*. At trial *t* response time is estimated by the time EA takes to reach a decision bound (i.e., the decision time *DT*) plus the non-decision time:

5 $${{RT}}_{t}={{DT}}_{t}+{\boldsymbol{nDT}}$$

Feedback driven learning was implemented by keeping a running average of the subjective expected value (also known as Q value) associated with each stimulus. Using a linear link function, we mapped the signed difference between stimulus values *Q*^*1*^ and *Q*^*2*^ to a (dynamic) drift rate (*DDR*), which was scaled by a parameter (*κ*) representing the subject-specific ability to modulate the SNR of decision evidence. The dynamic drift rate thus varied from trial to trial as a function of (learned) choice propensities. We modelled the white noise (*dW*) corrupting the decision process using a Gaussian distribution with mean 0 and variance *dt*. Bounded EA was updated at each time step *i* as per the following equation:

6 $${{EA}}^{i+1}={{EA}}^{i}+{\boldsymbol{\kappa }}{{DDR}}_{t}{dt}+{{dW}}^{i}$$

Based on this choice rule, in the case of an easy decision (i.e., the absolute difference between Q values is high), the average rate of evidence accumulation was high, resulting in more accurate and faster decisions. Conversely, as decision difficulty increased (i.e., the absolute difference between Q values is low), the average rate of evidence accumulation would drop, leading to less accurate and slower decisions. The average rate of evidence accumulation does in fact denote (and is inversely correlated with) decision difficulty. On average easy decisions tend to be faster and more accurate than difficult decisions. Furthermore, for any given level of decision difficulty, there is also a speed-accuracy trade-off. As decision time speeds up, accuracy drops and vice versa. In the context of diffusion models, this ubiquitous behavioural property is captured by the boundary separation parameter. We assumed an unbiased starting point (i.e. $\frac{a}{2}$) and symmetric decision bounds (*0* ≥ *EA*_*i*_ ≥ *a*).

The learning rule was a simple Rescorla-Wagner equation where the *Q* value of the chosen stimulus is updated as follows:

7 $${Q}_{t+1}={Q}_{t}+{\boldsymbol{\alpha }}({R}_{t}-{Q}_{t})$$

The difference between the expected *Q* value of the chosen stimulus and the observed feedback *R* is the reward prediction error (RPE). The learning rate *α* determines the extent to which the RPE is used to update the new *Q* value. We did not update the *Q* value of the unchosen stimulus. In total the hybrid RLDDM model has 4 free parameters (*nDT*, *κ, a* and *α*).

A major advantage of this hybrid modelling approach is that it describes both within-trial (i.e. processing of incoming decision evidence) and across-trials (i.e. feedback-based updating of the choice Q values) decision dynamics.

To probe the neural encoding of evidence integration we computed the area under the EA curves (Fig. 1B). As argued in previous work^32,36–39,42^, we reasoned that increasingly longer integration times result in an increasingly greater amount of integrated evidence (that is, greater area under the EA curves) and accumulator activity, which is reflected in greater BOLD responses (Fig. 1B). It is also worth noting that, as the temporal evolution of EA traces is shaped by the combined effects of multiple parameters, no simple one-to-one correspondence can be drawn between EA and single model parameters.

### Model fitting and validation

We fitted the hybrid RLDDM model to observed choice identities and latencies using a maximum a posteriori fitting routine. The objective function was the analytical approximation of the Wiener first passage time probability density function derived by Navarro and Fuss^65^ where the lower boundary represents correct model’s predictions:

8 $$f\left({{DT}}_{t}\right|{\boldsymbol{\kappa }}{{DDR}}_{t},\,{\boldsymbol{a}})$$

To preserve the parameters’ natural bounds, we implemented log (*a*) and logit (*α, nDT*) transforms of the parameters. For *nDT* we set an upper bound to each subject’s minimum RT so that *nDT* would never exceed it. We set the parameters’ (*κ, a*, *α, nDT*) prior means in their native space to (1,1,0,0.1) and their prior variances to 10.

To assess the model’s predictive performance (that is, the ability to predict observed behavioural effects^66^), we correlated observed mean choice accuracy and RT with the model’s predictions. While we calculated predicted choice accuracy based on the sign of the fitted dynamic drift rate, we estimated predicted mean RT using the following analytical solution^18^:

9 $$\overline{R{T}_{t}}=\frac{1}{2}\frac{{\boldsymbol{a}}}{{{\boldsymbol{\kappa }}{DDR}}_{t}}tanh\left(\frac{1}{2}{\boldsymbol{a}}{{\boldsymbol{\kappa }}{DDR}}_{t}\right)+{\boldsymbol{nDT}}$$

Moreover, to evaluate the model’s generative performance (that is, the ability to reproduce observed behavioural effects^66^) we first generated choice and RT data by running 5000 model simulations per subject using the fitted parameter estimates. We averaged observed and simulated behavioural data over each of the two choices and subsequently correlated these averages across subjects using 10% bend correlation^67^. Finally, to assess the robustness of the fitting procedure we performed parameter recovery^68^ and ascertained the strength of correlation between true and recovered parameter estimates.

### Analysis of RLDDM parameter estimates

To probe between-group differences of pre-treatment RLDDM parameter estimates we conducted the following analysis of covariance:

10 $${pe}=1+{response}+{preBDI}$$

where *pe* stands for parameters estimate, *response* is a categorical variable denoting treatment response and *preBDI* is a nuisance covariate representing pre-treatment BDI-II scores.

To evaluate any significant changes in the parameter estimates as a function of treatment, we fitted the hybrid RLDDM computational model to post-cCBT behavioural data. As we were primarily interested in assessing how the mean difference in pre-and post-treatment parameter estimates differed between responders and nonresponders after adjusting for pre-treatment depression severity, we ran the following analysis of covariance:

11 $$\Delta {pe}=1+{response}+{preBDI}$$

where $\Delta {pe}$ represents the difference between pre- and post-treatment parameter estimates.

### fMRI data acquisition and preprocessing

We recorded neuroimaging data using a 3T GE (General Electric, Milwaukee, Wisconsin) system with an eight-channel parallel imaging head coil. Neuroimaging acquisition parameters and preprocessing steps were previously documented^12^ and are reported again in the Supplemental Information.

### fMRI univariate analysis – pre-treatment between-group comparison

As the primary goal of our fMRI analysis was to determine whether pre-treatment accumulator-like haemodynamic activity was linked to cCBT response, we mainly focused on the task’s decision phase, which corresponds to the presentation of the two stimuli at the beginning of each trial. To this end, we first probed differential activations linked to decision evidence integration as a function of cCBT response (GLM1). We performed whole-brain statistical analyses using a multilevel mixed-effects approach as implemented in FLAME1 (FSL). At the first-level analysis, the regressor of interest was a model-derived trial-wise summary estimate of integrated decision evidence obtained by averaging the area under the accumulation curves generated through 5000 model simulations. We reasoned that smaller amounts of integrated evidence due to a steeper build-up of accumulator-like neuronal activity would produce smaller fMRI responses and vice versa. Moreover, the use of a model-derived parametric EA regressor afforded enhanced sensitivity to detecting accumulator-like activity in the brain. Three nuisance regressors accounted for visual stimulation, motor response and subjective valuation. All regressors were stick functions aligned with the onset of the decision phase. While the motor response regressor was modulated by choice RTs and the subjective valuation regressor was modulated by the absolute difference of the fitted Q values, the visual stimulation regressor was unmodulated. The design matrix also included three regressors modelling the outcome phase: two unmodulated stick functions capturing visual stimulation and late responses respectively and one modulated stick function representing the signed RPE. We convolved all regressors with a double gamma hemodynamic response function.

At the second-level analysis, we run an unpaired two-sample t-test to assess between-group differences of EA neural signatures. To explain away any between-group differential activations primarily driven by pre-treatment depression severity, we included pre-treatment BDI-II as a regressor of no interest. We thresholded the resulting z statistic images using a cluster-defining threshold of Z > 2.57 and an FWE-corrected significance threshold of *p* = 0.05. Given the relatively small sample size of this study, we chose a cluster-forming threshold of Z > 2.57 as it provides a compromise between adequately controlling false positive rate and improving sensitivity to less spatially extended activations (thus optimising statistical power).

### fMRI univariate analysis – conjunction overlay of pre-treatment mean and differential EA activity

To further refine detection of brain activations that scaled with evidence accumulation and differentiated between responders and nonresponders prior to cCBT, we first conducted a group-level one-sample t-test of the subject-wise linear contrast images representing integrated decision evidence and identified haemodynamic signatures of accumulator dynamics (GLM2). Subsequently, using the 3dcalc AFNI’s step-function, we performed a conjunction overlay of the group-level z-statistical images (thresholded at Z = 2.3) representing mean (GLM2) and (between-group) differential (GLM1) EA neural signatures and selected the top two largest conjunction clusters. The purpose of this analysis was to identify voxels with maximum effect size across the whole group mean and between-group EA activation maps.

### fMRI univariate analysis – brain-behaviour pre-treatment correlations

Due to the number of dropouts in the nonresponders group, we performed a supplementary fMRI analysis to test for a linear relationship between significant cluster-wise accumulator-like activity and clinical outcome. While we acknowledge the potential for inflated correlation estimates due to issues of circularity^69^, we conducted this analysis as a sanity check to safeguard against erroneous assumptions on the dropout group. We extracted the average beta weights from the pre-treatment EA clusters (GLM1) and conjunction clusters (GLM1 + GLM2) to test for a significant association with the magnitude of cCBT response as measured by the post-treatment residualized BDI-II scores (that is, after partialling out the variance associated with pre-treatment BDI-II scores). In addition to this analysis, in the nonresponders group, we also run a two-sample *t*-test of the cluster-wise average beta weights between dropouts and nondropouts.

### fMRI univariate analysis – brain-behaviour pre-post treatment correlations

Another goal of the fMRI analysis was to examine how EA neural signatures changed as a function of observed symptomatic improvement. The purpose of this analysis was to test whether cluster-wise pre-treatment activity was merely a biomarker of treatment response, or whether CBT treatment gave rise to a meaningful change in those brain areas. Due to the unbalanced repeated-measure dataset, we tested for a relationship between change in brain activity and symptomatic improvement. We first analysed post-treatment fMRI data using the same design matrix as for pre-treatment fMRI data. Then, using the pre-treatment EA clusters (GLM1) and conjunction clusters (GLM1 + GLM2), we correlated the difference between pre-and post-treatment cluster-wise beta weights with the magnitude of symptom change.

We performed all brain-behaviour correlations using robust 10% bend correlation, which protects against marginal outliers^67^ and corrected *p*-values for multiple comparisons using the Benjamini and Hochberg procedure^70^.

### fMRI multivariate analysis

To ascertain whether haemodynamic activity related to evidence accumulation reliably classified individual cCBT response we run a multivariate classification analysis of the individual spatially normalised first-level contrast images encoding integrated evidence.

We removed nonbrain voxels from the spatially normalized contrast images using a standard brain mask and scaled the data using feature-wise (i.e., column-wise) standardization. We run a linear L2-regularised/L2-loss support vector classifier from the open-source software liblinear^71^. We set hyperparameter C to the default value of 1. We implemented a 5-fold cross-validation scheme and randomly partitioned on subjects using the *cvpartition* function in MATLAB statistical toolbox (www.mathworks.com). To evaluate the classifier’s performance, we computed the area under the receiver operating characteristics curve (AUC-ROC) using the trapezoidal rule. To test statistical significance, we repeated cross-validation 100 times randomly shuffling the test set labels and estimated the proportion of results that were greater or equal to the observed classifier’s performance.

## Results

### Sample

Demographic and clinical characteristics of the recruited sample were reported in a previous paper^12^. Of the total 37 participants, only 26 subjects (~70%) completed cCBT and attended the post-treatment appointment. Of the 11 participants who did not attend the post-treatment appointment, 5 subjects did not complete cCBT due to lack of efficacy and 6 subjects deteriorated and required treatment with antidepressant medications. One subject was unable to undergo post-treatment scanning. In total, 19 subjects were classified as responders and 18 subjects were classified as nonresponders^12^. The overall cCBT response rate was 51.3%, which is consistent with expected response rates reported in the literature^72^. As already documented pre-treatment depression severity measured by BDI-II scores was significantly greater in the nonresponders than the responders’ group (*t*_35_ = 2.86, *p* = 0.006)^12^. Neither age (*t*_35_ = 0.08, *p* = 0.93) nor sex (${\chi }_{1}^{2}=0.02$, *p* = 0.86) significantly differed between responders and nonresponders.

### Behavioural results

We did not find any significant between-group differences in choice accuracy (β = 0.005, *p* = 0.96) and RTs (β = −0.021, *p* = 0.72) before cCBT (Fig. 1C, D). Moreover, there were no significant pre- to post-CBT differences in RTs (β = 0.024, *p* = 0.83) nor in choice accuracy (β = −0.04, *p* = 0.14). Likewise, neither RTs (β = −0.024, *p* = 0.72) nor choice accuracy (β = 0.18, *p* = 0.14) significantly changed as a function of cCBT response (Supplementary Fig. S1).

Overall, positive feedback speeded up RTs (β = −0.019, *p* = 0.035) but not as a function of CBT response (β = 0.002, *p* = 0.907). Likewise, correct choices were faster, on average, albeit not significantly (β = −0.012, *p* = 0.07) nor as a function of cCBT response (β = 0.004, *p* = 0.66).

### Model fit and parameter estimates

**Fig. 2:**
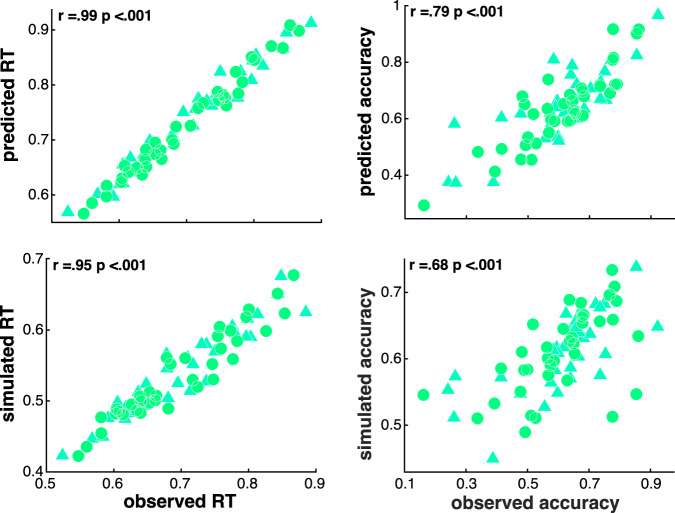
Predictive and generative performance of the hybrid RLDDM model. Observed subject- and choice-wise mean RT and accuracy were significantly correlated with model’s predicted and simulated data as shown in the scatterplots of this figure. r denotes the 10% bend correlation coefficient.

As shown in Fig. 2 our hybrid RLDDM model’s behavioural predictions and simulations significantly correlated with observed behavioural effects. Moreover, recovered parameters significantly correlated with the true data-generating parameters thus validating the fitting procedure used for parameter estimation (Supplementary Fig. S2).

**Fig. 3:**
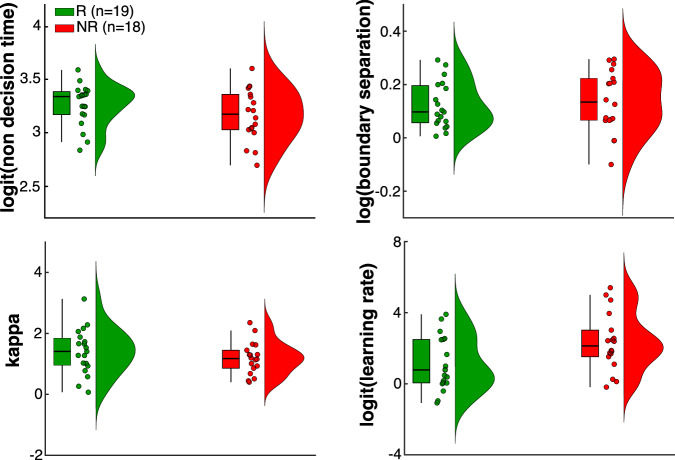
RLDDM parameter estimates. Raincloud plots showing pre-cCBT between-group comparisons of the parameter estimates in their native space. Boxplots display median and interquartile range. Colour-coded dots represent responders (R, green) and nonresponders (NR, red).

We did not find any significant pre-cCBT between-group differences of the fitted parameter estimates (non-decision time [*nDT*]: *t*_34_ = 1.13, *p* = 0.27; boundary separation [*a*]: *t*_34_ = 0.46, *p* = 0.65; kappa [*κ*]: *t*_34_ = 0.42, *p* = 0.67; learning rate [*α*]: *t*_34_ = −1.87, *p* = 0.07) (Fig. 3). Moreover, there was no significant change of the parameter estimates as a function of treatment response (non-decision time [*nDT*]: *t*_23_ = 0.93, *p* = 0.36; boundary separation [*a*]: *t*_23_ = −1.23, *p* = 0.23; kappa [*κ*]: *t*_23_ = −0.54, *p* = 0.60; learning rate [*α*]: *t*_23_ = −0.64, *p* = 0.53) although it is worth noting that the size of the nonresponders’ group after cCBT decreased due to dropouts (Supplementary Fig. S3).

### fMRI univariate analysis – pre-treatment between-group comparison

**Fig. 4:**
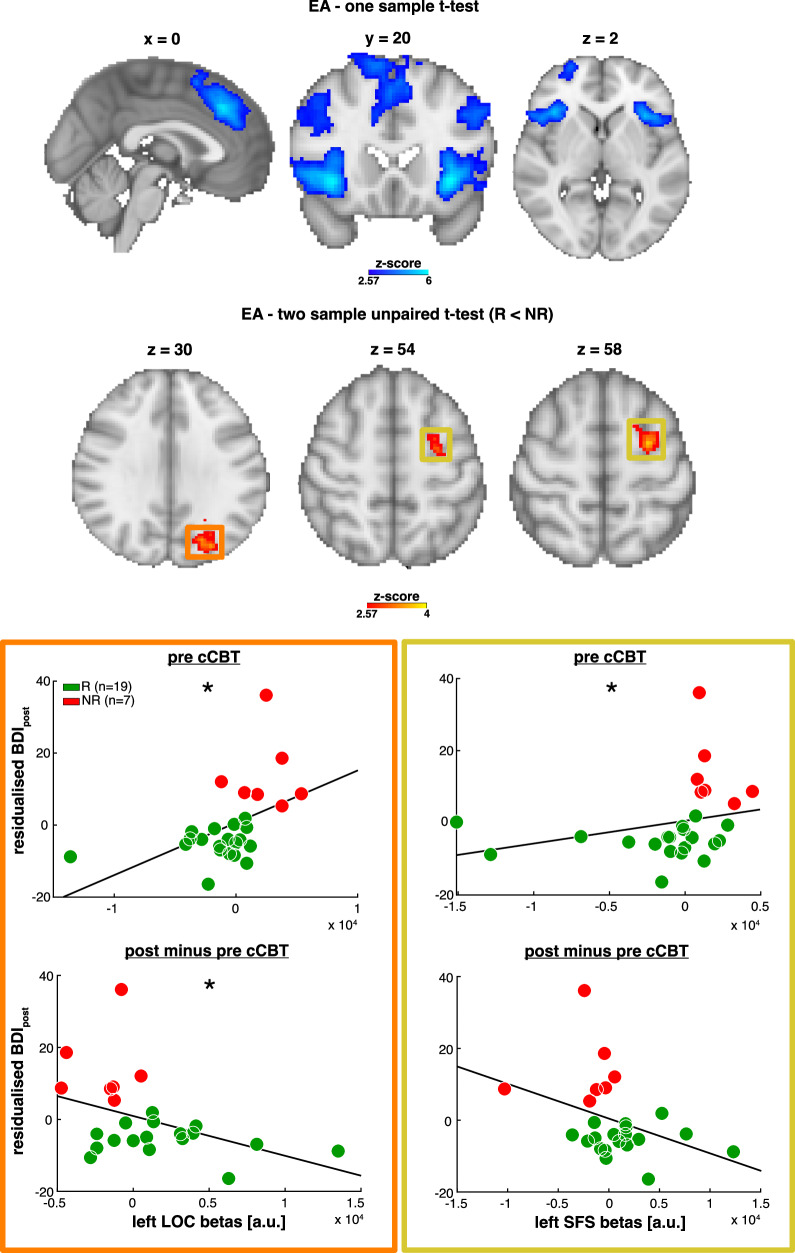
Average and differential (R < NR) pre-cCBT fMRI activity supporting evidence integration. Top panel. Average EA fMRI signatures (blue-light blue) in the bilateral medial prefrontal cortex (peak Z score = 5.67; MNI space coordinates = 0, 32, 38; *p* < 0.05 FWE), right lateral parietal and (superior) occipital cortex (peak Z score = 4.57; MNI space coordinates = 52, 48, 40; *p* < 0.05 FWE), right middle/superior frontal gyrus (peak Z score = 3.99; MNI space coordinates = 38, 30, 38; *p* < 0.05 FWE), right insula (peak Z score = 5.8; MNI space coordinates = 36, 20, −6; *p* < 0.05 FWE), left insula (peak Z score = 5.98; MNI space coordinates = −34, 20, −4; *p* < 0.05 FWE), left lateral parietal and (superior) occipital cortex (peak Z score = 4.58; MNI space coordinates = −40, −56, 48; *p* < 0.05 FWE), left middle frontal gyrus (peak Z score = 3.97; MNI space coordinates = −48, 18, 34; *p* < 0.05 FWE) and right frontal pole (peak Z score = 3.91; MNI space coordinates = 26, 64, 0; *p* < 0.05 FWE). Middle panel. Differential (R < NR) EA fMRI signatures (red-yellow) in the left lateral (superior) occipital cortex (LOC: peak Z score = 3.6; MNI space coordinates = −30, −88, 22; *p* < 0.05 FWE) (orange box), left superior frontal sulcus (SFS: peak Z score = 3.64; MNI space coordinates = −30, 0, 62; *p* < 0.05 FWE) (yellow box) and left cerebellum (peak Z score = 3.79; MNI space coordinates = −10, −40, −26; *p* < 0.05 FWE) (not shown here). All fMRI statistical maps are FWE-corrected (Z = 2.57). Bottom panel. We extracted the pre-cCBT beta weights from the left LOC/SFS clusters and correlated them with post-cCBT residualised BDI scores (lower scores indicate greater clinical improvement) as shown in the top scatterplots in the orange (left LOC) and yellow (left SFS) boxes. We also estimated the difference between pre- and post-cCBT beta weights extracted from the left LOC/SFS and correlated them with post-cCBT residualised BDI scores as shown in the bottom scatterplots. Solid black lines represent least-square linear fits. Colour-coded dots represent responders (R, green) and nonresponders (NR, red). *p < 0.05.

While we did not observe any reliable differential patterns in the behavioural performance, we wanted to test whether looking in the latent space of neural data would afford us additional explanatory power, as some of the between-group effects might be too subtle to manifest in behaviour. More specifically, we probed the neural correlates of evidence integration as a function of cCBT and found EA signatures in a distributed fronto-parieto-occipital network and the bilateral insula (Fig. 4). Based on increasing evidence that efficiency of evidence integration represents a transdiagnostic vulnerability factor in mental disorders^43,53^, we chose to investigate whether EA signatures may account for differential response to cCBT.

We found that, at baseline, compared to nonresponders, responders showed significantly weaker BOLD activity covarying with integrated evidence in the left superior frontal sulcus (SFS), left lateral occipital cortex (LOC) and left cerebellum (Fig. 4). It is worth noticing again that the observed EA-related differential brain activations are independent of baseline depression severity as the baseline BDI-II scores were included in the analysis as nuisance regressor. We did not find any significantly greater activations in the responders group compared to nonresponders. Likewise, we did not find any significant activations covarying with baseline depression severity.

### fMRI univariate analysis – conjunction overlay of pre-treatment mean and differential EA activity

**Fig. 5:**
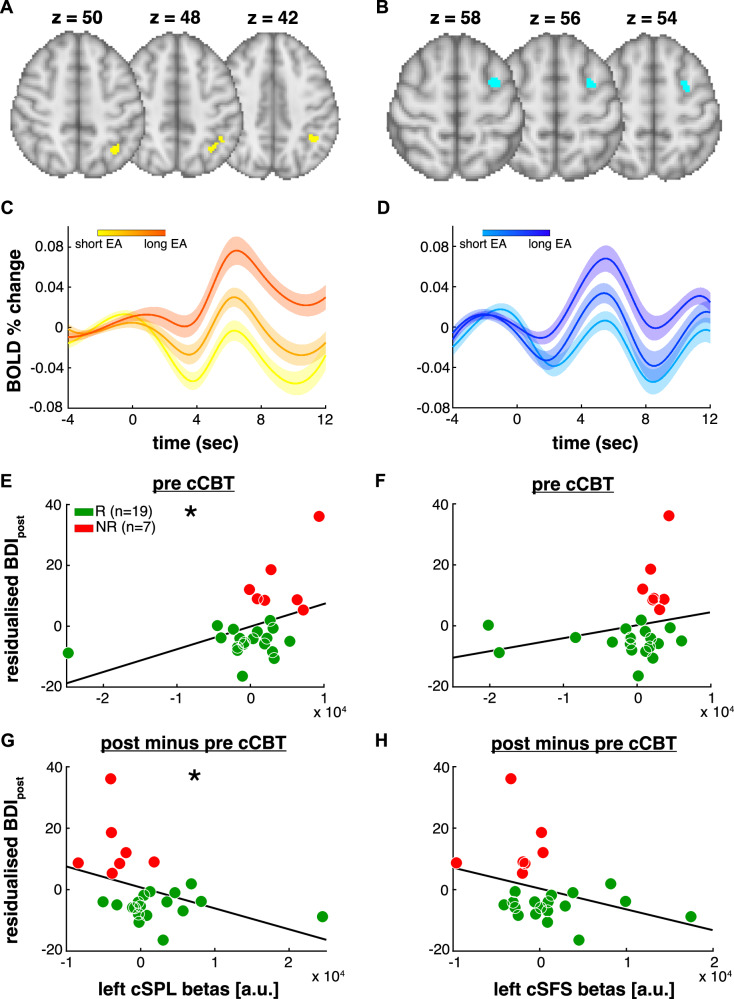
EA conjunction overlay. **A**, **B** We performed a conjunction overlay of the average and differential (R < NR) statistical maps capturing accumulator-like activity. The two largest clusters were in the left superior parietal lobule (cSPL; in **A**) and left superior frontal sulcus (cSFS; in **B**). **C**, **D** We retrieved the conjunction cluster-wise BOLD percent signal change traces. We sorted trials based on the tertiles of the model-derived EA estimates and plotted the average BOLD percent signal change traces for each tertile to demonstrate the monotonic effect of evidence integration on the cluster-wise haemodynamic responses from the regions shown in (**A**) and (**B**) respectively. **E**, **F** We extracted the cluster-wise beta weights and plotted them as a function of the post-cCBT residualised BDI scores from the regions shown in (**A**) and (**B**) respectively. **G**, **H** We computed the difference between the pre- and post-cCBT beta weights extracted from the two conjunction clusters shown in (**A**) and (**B**) and plotted them as a function of the post-cCBT residualised BDI scores. Solid black lines represent least-square linear fits. Colour-coded dots represent responders (R, green) and nonresponders (NR, red). *p < 0.05

The two largest clusters resulting from the conjunction overlay of group-level mean and differential EA statistical maps were in the left SFS (79 voxels) and left superior parietal lobule (SPL) (116 voxels) (Fig. 5A, B). An exhaustive list of the conjunction clusters is presented in the Supplementary Table S1.

### fMRI univariate analysis – brain-behaviour pre-treatment correlations

Subject-wise beta weights extracted from the left SFS and LOC EA clusters (GLM1) significantly correlated with post-cCBT residualised BDI-II scores (left SFS: *r*_35_ = 0.49., *p*_adj_ = 0.045; left LOC: *r*_35_ = 0.58, *p*_adj_ = 0.014). In the nonresponders group there was no significant difference of cluster-wise EA-related brain activity between dropouts and nondropouts (left SFS: *t*_16_ = −0.38, *p* = 0.71; left LOC: *t*_16_ = −0.56, *p* = 0.58).

As shown in Fig. 5E, F subject-wise beta weights extracted from the conjunction clusters (GLM1 + GLM2) significantly correlated with the magnitude of post-cCBT symptomatic improvement in the left SPL_conj_ (r_35_ = 0.41, *p*_adj_ = 0.050) but not the left SFS_conj_ (*r*_35_ = 0.39, *p*_adj_ = 0.094). Moreover, in the nonresponders group, there was no significant difference of cluster-wise EA-related brain activity between dropouts and nondropouts (left SFS_conj_: *t*_16_ = −0.42, *p* = 0.68; left SPL_conj_: *t*_16_ = 0.02, *p* = 0.98).

### fMRI univariate analysis – brain-behaviour pre-post treatment correlations

We found that the subject-wise differences between pre- and post-cCBT beta weights significantly correlated with clinical improvements in the left LOC (*r*_23_ = −0.45, *p*_adj_ = 0.045) and left SPL_conj_ (*r*_23_ = −0.47, *p*_adj_ = 0.045) but not in the left SFS (*r*_23_ = −0.40, *p*_adj_ = 0.071) and left SFS_conj_ (*r*_23_ = −0.35, *p*_adj_ = 0.094) (Fig. 5G, H).

### fMRI multivariate analysis

Subject-wise EA fMRI maps significantly classified individual cCBT response (AUC-ROC = 0.66, *p*_perm_ = 0.02). In other words, we found that pre-treatment EA-related neural activity yielded reliable out-of-sample treatment response classification.

In sum, attenuated activity of left-sided prefrontal and parieto-occipital accumulators was associated with response to cCBT and increased following cCBT commensurably with reduction of depression severity.

## Discussion

In this study we leveraged the neurocomputational signatures of value-based decision-making during a probabilistic reversal learning task, akin to a two-alternative forced-choice task under time pressure^73^. More specifically, we probed neural activity supporting the stochastic drift of decision evidence towards a decision boundary, known as bounded EA, as a function of response to cCBT. Critically, while we did not see any significant between-group difference in the behavioural performance, we found preliminary evidence of cCBT-related neural effects, highlighting the potential utility of neural signatures over more traditional behavioural or computational phenotyping.

Across the whole sample of unmedicated depressed subjects, we observed neural signatures of bounded EA in a distributed fronto-parieto-occipital network and the insula. More importantly, we showed that compared to nonresponders, responders exhibited significantly weaker EA signatures in the left SFS and left superior LOC. Crucially, the degree to which this accumulator activity in the left LOC increased following cCBT significantly correlated with symptomatic improvement. In essence, depressed subjects who showed lesser recruitment of neural accumulators at the time of deliberation had the greatest clinical improvement after cCBT.

The functional neuroanatomy of the identified cortical integrators is intriguing and consistent with extant literature. The two largest EA conjunction clusters corresponded to the left SFS and left SPL. Previous human fMRI^26^ and interventional^31^ studies characterised the left SFS as a higher-level decision-making area integrating the output from lower-level sensory processing regions to form a decision, independent of response modality^74,75^. Similarly, human neuroimaging studies showed that neural activity in the intraparietal cortex^32–35,38^ (human homologue of the lateral intraparietal [LIP] area identified by early pioneering work in nonhuman primates^20–23^) and occipital cortex^33,37^ reflected integration of perceptual information.

At the neural level, previous work showed that EEG signatures denoting post-sensory encoding of EA^41,76,77^ during a visual categorisation task were amplified over the course of perceptual training and correlated with enhanced behavioural performance^28,78^. In other words, greater accumulator activity in the brain fosters more efficient decision-making dynamics as inferred from observed improvements of choice behaviour. Furthermore, there is evidence that dopamine modulates the rate of EA by regulating the SNR of incoming decision evidence. Using different dosages of methylphenidate, a mixed dopamine/norepinephrine transporter blocker, in healthy subjects, Beste et al. demonstrated that lower dose methylphenidate significantly increased the drift rate as a function of motion coherence during a random dot motion task^79^. Another study found that increased dopamine bioavailability by means of levodopa administration to healthy adults altered the effects of cathodal transcranial direct stimulation on response discriminability during visual decision making^80^. While dopaminergic modulation directly influences neural activity in the prefrontal cortex via the mesocortical pathway, there is emerging evidence that dopamine also has downstream effects on the neural activity of the parietal^81^ and occipital^82^ cortex.

This potential role of dopamine in EA is intriguing since core depressive features such as anhedonia and apathy have been ascribed to dysfunctions in the dopamine system^83–85^. Similarly, compared to healthy controls, depressed patients’ reduced reward response bias in a probabilistic reward task^86^ was accounted for by a shallower EA slope, which predicted the reduced total sum of rewards obtained by depressed patients^45^. Using the same behavioural paradigm, a later study corroborated and extended this finding by showing that greater depression severity in a sample of late childhood and early adolescence girls was correlated with slower EA^47^. Furthermore, slow EA during positive memory retrieval^46,48^ gives rise to the negative memory bias commonly observed in depression^87^. Taken together, these findings convincingly implicate abnormal EA dynamics as a key dysfunction underlying the negatively biased cognitive distortions seen in depressed patients^88^.

Since CBT mainly works by helping patients identify and challenge negative cognitive biases in decision-making^6^, which empirical evidence has linked to sluggish EA dynamics, it is tempting to hypothesise that changes in EA-related neural activity may be a candidate target through which CBT exerts its therapeutic effects although further research is required to draw any causal inference in this regard. Consistent with our hypothesis is the observed increase of left prefrontal and parieto-occipital accumulator activity as a function of reduced depression severity. As we cannot conclusively attribute the observed neural effects to cCBT, a natural extension of our work would be to test whether attenuated EA signatures represent the neural substrate of a pre-existing decision-making deficit, which is remedied by cCBT^89^.

Contrary to the research work on depression outlined above, we fitted a hybrid RLDDM computational model where a dynamic drift rate is derived from the trial-by-trial difference of the Q values^62,63^, which are continuously updated based on explicit feedback. Therefore, in our model the dynamic drift rate incorporates a learning component, which is scaled by a fixed parameter (κ) representing the subject-specific sensitivity to the “learned” difficulty effects. In other words, as the Q value difference grows, choices become faster and easier. While in conventional DDM-only computational models such difficulty effects are predominantly captured by the drift rate parameter, in the hybrid RLDDM they are only partially reflected in the κ parameter, which may explain the lack of any significant cCBT-related effects. It is also possible that our study was not powered enough to detect behavioural and computational effects.

Importantly, the efficiency of EA processes is largely determined by domain-general, individual traits and only marginally influenced by task-specific (i.e. difficulty) and state-dependent (i.e. attention) factors^43^. While test-retest reliability of RL parameters is poor^90^, calling into question their utility for computational phenotyping, test-retest reliability of the drift rate (and other DDM parameters) is good^91^, casting the efficiency of EA processes as a robust psychometric measure of individual differences. As reduced EA efficiency accounts for neurocognitive impairments across a broad range of cognitive domains, Weigard et al. proposed it may serve as a “watershed node” linking an array of biological and contextual factors to general psychopathology^43^.

While we report a strong association and linear dose-response relation between BOLD activity of left prefrontal and parieto-occipital accumulators and clinical improvement, we cannot determine their specificity to cCBT^92^. It is possible that we have revealed a general and non-specific neural signature of depressive symptoms amelioration, which is independent of the CBT-based intervention. In accordance with this interpretation is the observation that the left dorsolateral prefrontal cortex, including the left SFS, is the site targeted by repetitive transcranial magnetic stimulation in the treatment of refractory depression^93^. In addition to treatment specificity, we cannot definitively ascertain whether the identified EA signatures are specific to depression or instead represent a global prognostic biomarker cutting across psychiatric diagnostic categories. In favour of this contention are the observations that EA i) correlates with transdiagnostic severity of general psychopathology^53^ and ii) also improves following stimulant treatment in ADHD^94^. While patients were clinically assessed at follow-up by a qualified psychiatrist to determine symptom resolution independent of CBT, further evaluation of depression severity immediately after treatment or even at multiple follow-up points would have made ascertainment of clinical response more robust.

In conclusion, in this study we have shown preliminary evidence that reduced efficiency of evidence integration in left prefrontal and parieto-occipital neural accumulators is associated with response to cCBT in unmedicated depressed patients. Intriguingly, clinical effectiveness of cCBT linearly scales with increased post-treatment EA fMRI signatures. We discuss our finding of reduced efficiency of decision dynamics in the context of the putative neurocognitive processes linked to CBT therapeutic effects in depression including limitations of our study and future directions for further experimental work.

## Supplementary information

Supplementary Information

## Data Availability

The code used for statistical and computational modelling will be made available upon request.
