# Supplementary material for: Prefrontal and parieto-occipital neural signatures of evidence accumulation and response to computerised Cognitive Behavioural Therapy in depression
Source: Npj Ment Health Res. 2025 Oct 14;4:51. doi: 10.1038/s44184-025-00165-3 (PMC12521554; doi:10.1038/s44184-025-00165-3)
Supplement: Supplementary file 1 — Supplementary Information [file 44184_2025_165_MOESM1_ESM.docx]

**Supplemental Information**

***The Glasgow CBT study***

The Glasgow CBT study is a research project whose aim was to identify candidate neuroimaging predictors of response to computerised CBT (cCBT) in unmedicated depressed subjects. Participants were recruited from the general population via an advert in a local newspaper and self-referred to the research team. Their eligibility was assessed by a qualified psychiatrist (FQ).

Inclusion criteria were a primary diagnosis of depressive disorder fulfilling the International Classification of Diseases (10^th^ Revision) diagnostic criteria; age between 16 and 65 years; a score of ≥14 on the BDI-II. Exclusion criteria were ongoing CBT or other psychological therapy; current prescription of psychotropic medication; history of brain injury; diagnosis of other major psychiatric disorder; history of CBT treatment in the previous 3 years; current diagnosis of substance misuse.

The study’s psychological intervention was an online CBT-based guided self-help program, “Living Life to the Full Interactive” (<http://llttf.com>), which was made freely available to the participants. Participants completed the CBT programme’s six online modules over a period between 6 and 10 weeks and received weekly telephone guidance from a support worker. Crucially, the use of cCBT abolished the confounding effect of therapist variability on treatment response and enabled monitoring of treatment adherence via the number of online modules completed.

A total of 48 subjects enrolled in the Glasgow CBT study and took part in a range of task-based and resting state fMRI measurements. Only 37 participants (18 females) from the Glasgow CBT study’s sample completed the experimental paradigm discussed in this paper.

***fMRI acquisition parameters***

We acquired a high-resolution T1-weighted structural image (0.5 mm x 0.5 mm x 1 mm voxels, 320 x 320 matrix, 160 axial slices, in- version time (TI) = 500 ms, repetition time (TR) = 7700 ms, echo time (TE) = 1.5 ms, flip angle (FA) = 12°) using an optimized inversion recovery fast spoiled gradient echo sequence and a functional echo planar imaging scan (3.75 x 3.75 x 4.5mm voxels, 64 x 64 matrix, 264 axial slices, TR = 2000 ms, TE = 35 ms, FA = 80°). Slice orientation was tilted to +20° from the anterior commissure-posterior commissure plane to alleviate signal dropout in the orbito-frontal cortex (Weiskopf et al., 2006). The first four volumes of the functional scan were discarded to allow for the magnetic field to reach the steady state.

***fMRI data preprocessing***

Functional MRI data were preprocessed and analysed using FSL (FMRIB’s software library) software. Preprocessing pipeline involved intramodal motion correction using MCFLIRT (motion correction FMRIB’s linear image registration tool), slice timing correction, spatial smoothing with an isotropic 5-mm full width at half maximum Gaussian kernel, high-pass temporal filtering with a cut-off frequency of 100 s, and grand-mean intensity normalization of each entire four-dimensional dataset. Functional scans were subsequently coregistered with skull-stripped structural images using boundary-based registration as implemented in FLIRT (FMRIB’s linear image registration tool) and spatially normalized into MNI152 space using FNIRT (FMRIB’s non-linear image registration tool) nonlinear registration.

***fMRI univariate analysis – brain-behaviour pre-post treatment correlations (2x2 mixed ANOVA)***

To test the additional hypothesis that post-CBT changes in the activity of the two pre-treatment EA clusters (GLM1) and two conjunction clusters (GLM1+GLM2) are significantly different between responders (R) and nonresponders (NR), we performed a 2x2 mixed ANOVA using the *rstatix* R package with session (baseline vs follow-up) as within-subjects factor and response (R vs NR) as between-subjects factor. We found that the session x response interaction effect was statistically significant for three of the four clusters (left LOC: F_1,23_ = 5.33, p = .03; left SFS: F_1,23_ = 5.62, p=.027; left SPL_conj_: F_1,23_ = 5.46, p = .029; left SFS_conj_: F_1,23_ = -4.12, p = .054). However, these results should be interpreted with caution due to the risk of bias and lack of reliability associated with the markedly unbalanced design (18 R and 7 NR), which significantly reduces statistical power.

**Supplementary Figures**


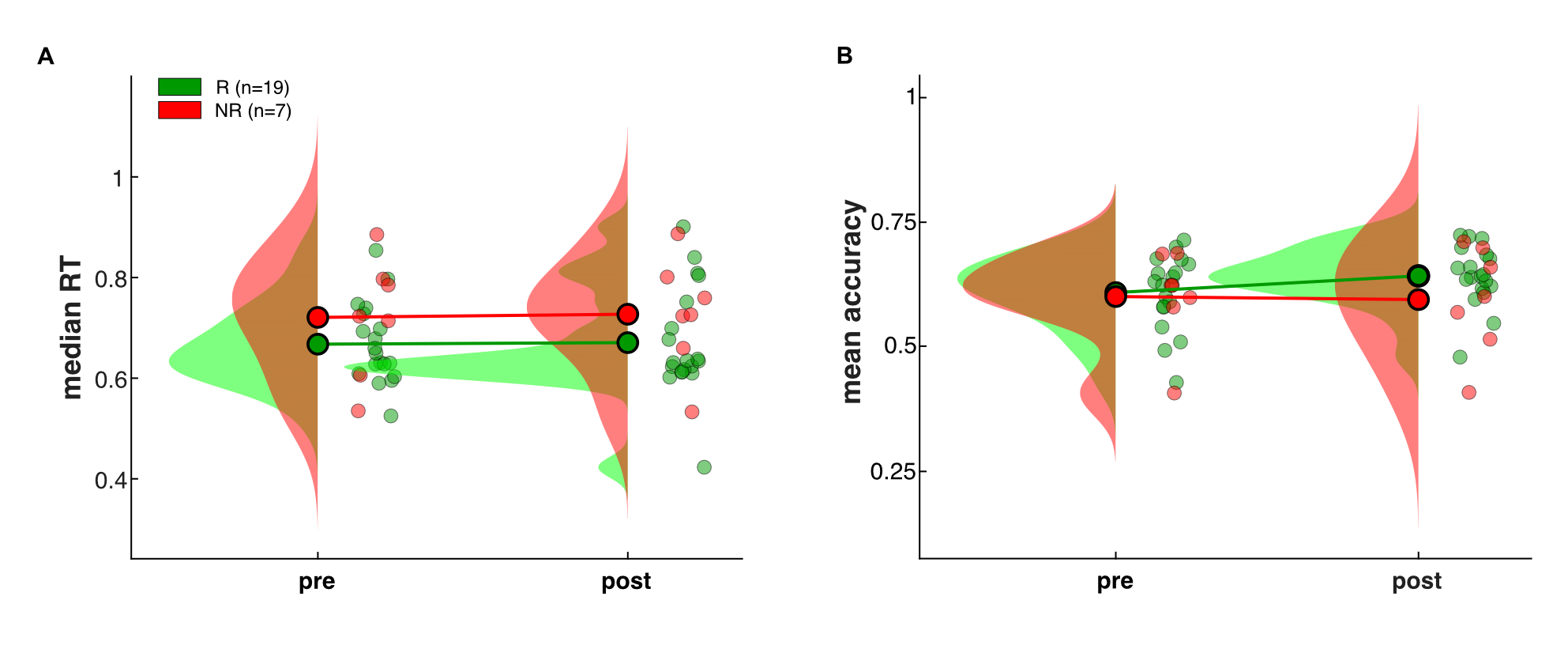


**Figure S1. Longitudinal change of behavioural performance.** (A-B) Raincloud plots showing pre- and post-cCBT changes in median RTs (A) and mean choice accuracies (B) as a function of treatment response. Colour-coded dots represent responders (R, green) and nonresponders (NR, red).

**Figure S2. RLDDM parameter recovery.** Scatterplots showing correlations between true and recovered parameter estimates. Correlations estimates greater than 0.5 are fair and those greater than 0.9 are excellent. r stands for 10% bend correlation coefficient. Dots show individual data points.

**Figure S3. Longitudinal change of RLDDM parameter estimates.** Raincloud plots showing pre- and post-cCBT changes in parameter estimates as a function of treatment response. Colour-coded dots represent responders (R, green) and nonresponders (NR, red).

**
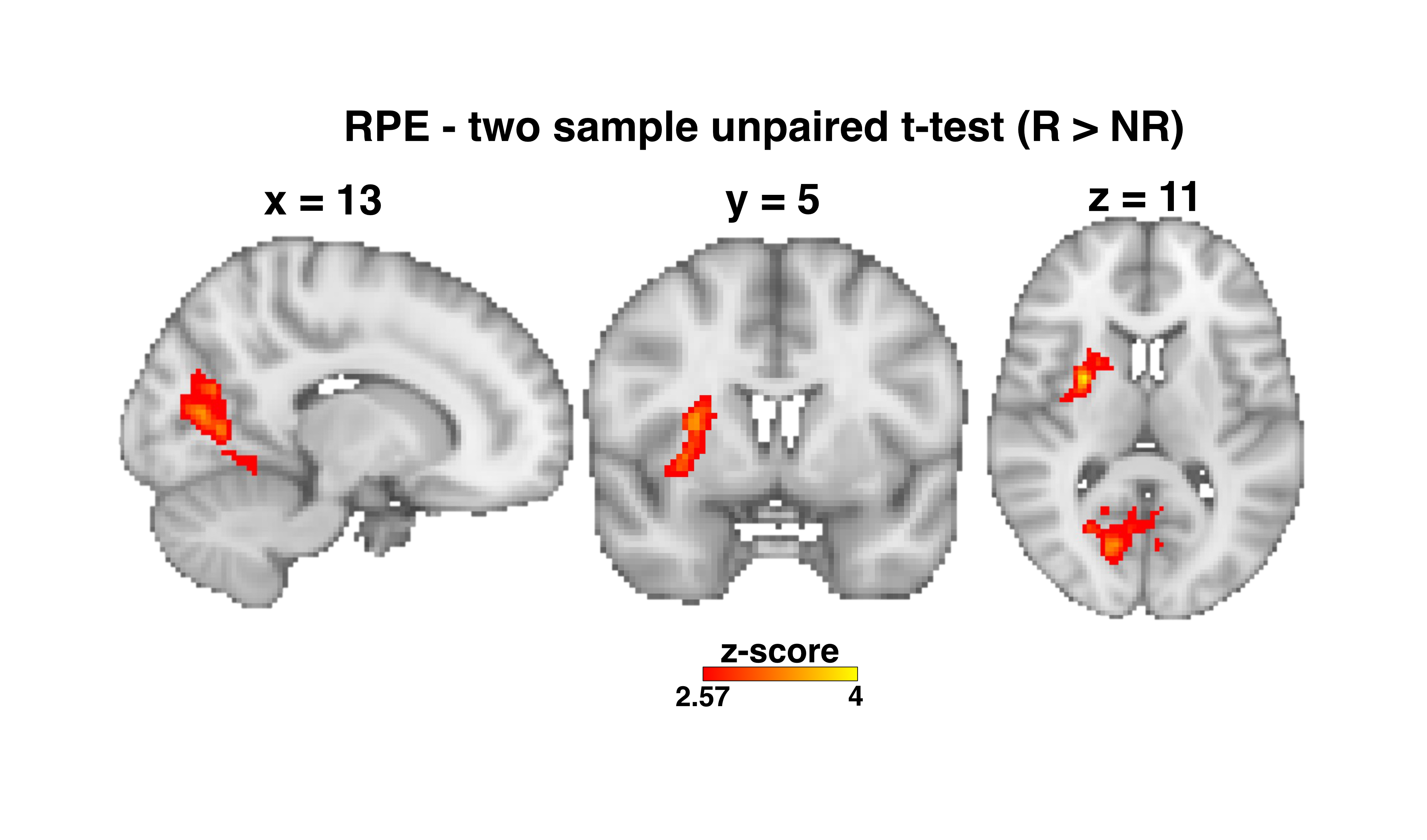
**

**Figure S4. Reward prediction error (RPE).** While the outcome phase was not the primary focus of the fMRI analysis, we found RPE-related haemodynamic responses to be greater in the right putamen (peak Z score = 3.84; MNI space coordinates = 28, 2, 12; p < .05 FWE) and right intracalcarine cortex (peak Z score = 3.38; MNI space coordinates = 16, -74, 12). for the responders compared to the nonresponders group, thus confirming previously reported results (Queirazza et al., 2019), albeit obtained with a different computational model.

| **EA Conjunction Cluster** | **Number of Voxels** |
| --- | --- |
| Left Superior Parietal Lobule | 116 |
| Left Superior Frontal Sulcus | 77 |
| Left Cerebellum | 44 |
| Right Lateral Occipital Cortex | 43 |
| Right Middle Frontal Gyrus | 39 |
| Right Precentral Gyrus | 37 |
| Left Frontal Pole | 36 |
| Left Lateral Occipital Cortex | 19 |
| Right Precuneus Cortex | 15 |
| Right Angular Gyrus | 12 |

**Table S1. EA conjunction clusters.** Clusters were obtained from the conjunction overlay of mean and differential (R<NR) statistical z-maps (thresholded at Z = 2.3) denoting evidence accumulation (EA). Only clusters with > 10 voxels are shown.

**References**

Fan R-E, Chang K-W, Hsieh C-J, Wang X-R, Lin C-J (2008) LIBLINEAR: A Library for Large Linear Classification. J Mach Learn Res 9:1871–1874.

Queirazza F, Fouragnan E, Steele JD, Cavanagh J, Philiastides MG (2019) Neural correlates of weighted reward prediction error during reinforcement learning classify response to cognitive behavioral therapy in depression. Sci Adv 5:eaav4962.

Weiskopf N, Hutton C, Josephs O, Deichmann R (2006) Optimal EPI parameters for reduction of susceptibility-induced BOLD sensitivity losses: a whole-brain analysis at 3 T and 1.5 T. Neuroimage 33:493-504.
